# Case report: Primary intraosseous meningioma: a radiological study of two cases confirmed pathologically

**DOI:** 10.3389/fonc.2025.1502818

**Published:** 2025-02-04

**Authors:** Yue Wang, Jibo Hu

**Affiliations:** Department of Radiology, The Fourth Affiliated Hospital of School of Medicine, and International School of Medicine, International Institutes of Medicine, Zhejiang University, Yiwu, China

**Keywords:** meningioma, intraosseous, pathology, diagnosis, case report

## Abstract

**Introduction:**

Primary intraosseous meningioma (PIM) is a rare lesion often misidentified preoperatively due to its ambiguous benign or malignant characteristics. In this report, we introduce two novel cases of PIM and explore the potential correlation between pathological classification and imaging features. Our aim is to enhance our understanding of PIM and improve its preoperative diagnosis.

**Case presentation:**

The first case is a 68-year-old female patient presenting with a brain mass located in the temporal region. Computed tomography (CT) imaging demonstrated the destruction of adjacent bone structures. A right frontal temporal craniectomy was subsequently performed and histological examination pathologically confirmed the lesion was the chordoid variant of PIM. The second case is a 56-year-old male patient who exhibited an irregular soft-tissue mass in the right sphenoid as visualized on brain CT. The patient underwent a surgical intervention for a skull base neoplasm. Postoperative pathological analysis confirmed the presence of the meningothelial variant of PIM. Upon pathological examination, the two cases were respectively classified as atypical meningioma (Grade II) and benign meningioma (Grade I).

**Conclusions:**

While pathological examination remains indispensable for the definitive confirmation of PIM, the early identification of PIM is critically dependent on radiological imaging methods. The imaging characteristics of PIM exhibit variability across different pathological grades, a factor that can significantly aid in both the diagnostic process and the formulation of appropriate treatment strategies.

## 1 Introduction

Primary extradural meningiomas (PEMs) constitute less than 2% of meningiomas overall (1). Within this subset, the incidence of primary intraosseous meningiomas (PIMs), which originate from intraosseous locations, has been estimated to account for two-thirds of PEMs (2). The World Health Organization (WHO) classification system for meningiomas, established in 2007, categorizes these tumors into three distinct grades: benign meningioma (Grade I), atypical meningioma (Grade II), and anaplastic meningioma (Grade III). The options for treatment and the prognosis of the three grades are significantly different, highlighting the importance of accurate classification (3).

Computed tomography (CT) and magnetic resonance imaging (MRI) are invaluable tools for assessing the status of calvarial lesions (4). Osteolytic skull lesions accompanied by soft-tissue masses are more likely to be indicative of malignant meningiomas (4). Herein, we present the imaging findings of PIMs in two patients with different pathological grades to further elucidate the diagnostic challenges and potential clinical implications associated with these rare tumors.

## 2 Patients and methods

### 2.1 Patients

We conducted a retrospective review of two patients diagnosed with PIMs who were admitted and treated at our institution. This study was approved by the Human Research Ethics Committee of the Fourth Affiliated Hospital of Zhejiang University School of Medicine, ensuring adherence to ethical standards and patient confidentiality.

## 3 Case presentation

### 3.1 Case 1

**Figure 1:**
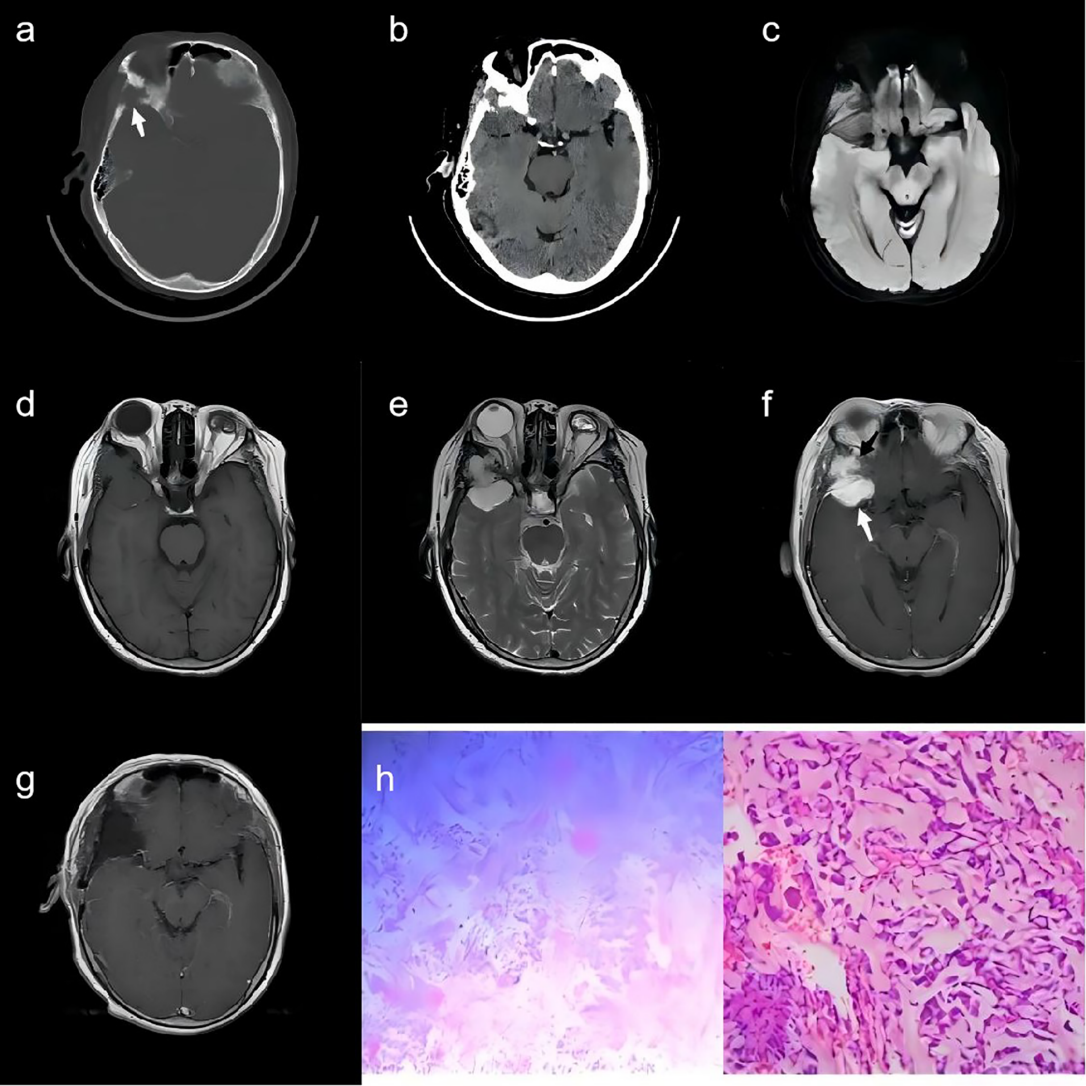
A 68-year-old woman. **(A)** A CT scan with bone window demonstrates the anterior part of the right temporal bone destruction (white arrow). **(B)** A Brain CT scan shows a rounded-like soft-tissue density mass in the temporal region. **(C–E)** MR images depict a dumbbell-shaped intradiploic mass that was iso-hypo signal intensity on T1WI **(D)** and diffusion sequence **(C)**, and hyperintensity on T2WI **(E)**. **(F)** The lesion shows intense and heterogeneous enhancement in two parts (white arrow and black arrow, respectively) on contrast-enhanced T1WI. **(G)** The post-operative contrast-enhanced T1WI revealed no significant enhancement in the lesion region. **(H)** Pathological examination revealed chordoid meningioma (CNS WHO grade II) with eosinophilic vacuolated tumor cells on a myxoid background.

A 68-year-old woman was admitted to the hospital due to intermittent pain on the right side of her head and neck, which had been present for the past year and had worsened over the previous 3 months. She had no family history of hereditary diseases or tumors. Upon physical examination, it was noted that her right eye was protruding. The neurological examination was negative. A brain CT scan revealed a rounded-like soft-tissue density mass in the temporal region, expanding the anterior part of the right temporal bone and the lateral orbital wall. This mass was associated with significant bone destruction and hyperostotic changes in the surrounding bone (**Figures 1A, B**). An MRI was promptly conducted that showed a dumbbell-shaped intradiploic mass. This mass appeared as an iso-hypo signal intensity on T1-weighted images (T1WIs) and diffusion sequence (**Figures 1C, D**) and as a hyperintense signal on T2-weighted images (T2WIs) (**Figure 1E**). It also demonstrated prominent and heterogeneous enhancement on contrast-enhanced T1WI. Additionally, gadolinium enhancement of the underlying dura was also observed (**Figure 1F**). Based on these findings, the preoperative diagnosis was either a temporal bone meningioma or a solitary fibrous tumor.

Following a collaborative assessment by radiologists and neurosurgeons, it was determined that the lesion in the right middle cranial fossa was in close proximity to the anterior branch of the right middle cerebral artery. Consequently, it was decided to perform preoperative endovascular embolization of the dura mater for the patient. On the second day, a right frontotemporal craniotomy was performed. The tumor was then excised to Simpson’s grade 1. Intraoperatively, it was discovered that the tumor had breached the dura mater, infiltrating the outer wall of the orbit, the sphenoid bone, and the temporalis muscle. The deep aspect of the tumor was meticulously dissected, and the involved skull bone of the outer orbital wall was drilled and removed. To reconstruct the skull, cranioplasty was performed using three sets of Kangtuo connecting plates. On the third day following the surgery, the patient underwent a postoperative MRI scan. The results revealed no significant enhancement in the lesion region, suggesting that the surgical procedure had successfully achieved a complete resection of the tumor, and there was no indication of residual disease (**Figure 1G**).

Postoperatively, the patient developed an intracranial infection. This was promptly addressed with antimicrobial therapy, and regular lumbar punctures were performed during follow-up to monitor the patient’s condition and to manage any potential complications related to the infection. After an additional 3 weeks of postoperative care, the patient was discharged with restored normal inflammatory markers, indicating that the infection had been successfully treated. During the 1-month follow-up period, there was no recurrence of the tumor, which is a positive sign for the patient’s long-term prognosis.

Pathological examination indicated that, within the mucinous stroma, the tumor cells were predominantly arranged in a spindle-shaped pattern, with some arranged in whorls. Eosinophilic vacuolated cells and minimal cellular anisocytosis were present. Interstitial vascular proliferation was observed, accompanied by chronic inflammatory cell infiltration in the area. Immunostaining for epithelial membrane antigen (EMA) was positive within the cytoplasm of the neoplastic cells (**Figure 1H**).

Histologically, the tumor was diagnosed as a primary intraosseous chordoid meningioma, classified as WHO Grade II.

### 3.2 Case 2

**Figure 2:**
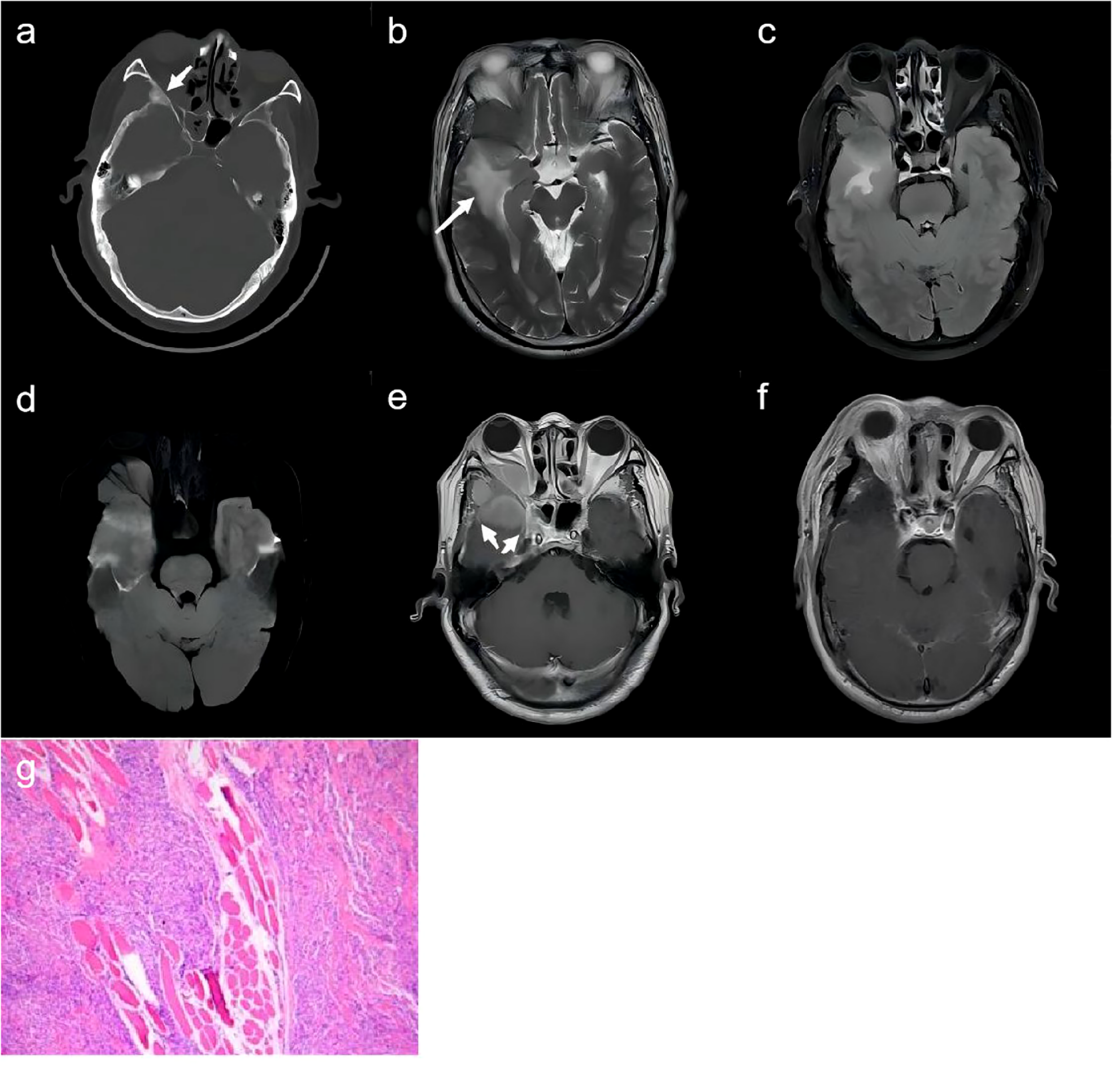
A 56-year-old man. **(A)** CT scan with bone window demonstrates the spheno-orbital osteocondensing lesion (white arrow). **(B–D)** MR images depict the right spheno-fronto-temporo-orbital mass with edema (white arrows) of the surrounding brain parenchyma which showed hyperintensity on T2WI **(B)**, T2-weighted fluid-attenuated inversion recovery **(C)**, and diffusion sequence **(D)**. **(E)** The lesion shows homogeneous enhancement with involved meningeal enhancement (white arrows) on postcontrast T1-weighted. **(F)** The post-operative enhanced MRI scans revealed patchy enhancement within the region that was previously occupied by the lesion. **(G)** Pathological examination revealed meningothelial meningioma (CNS WHO grade I) with epithelioid tumor cells.

A 56-year-old male patient was referred to the hospital after experiencing a sudden loss of consciousness 2 days prior to admission. Upon examination, he exhibited limited abduction of the right eye, noticeable visual field defects in the right eye, and scattered dark spots in the left eye. Neurological examination revealed no abnormalities. The patient had a history of hypertension for over 5 years and had been a smoker for 30 years, consuming approximately 20 cigarettes daily. Additionally, he reported moderate alcohol consumption for 30 years, which did not affect his daily life or work. Brain CT demonstrated an irregular soft-tissue mass in the wing of the sphenoid on the right side, partially extending into the middle cranial fossa (**Figure 2A**). The mass was poorly defined from the temporal lobe, accompanied by edema in the surrounding brain parenchyma. It also extended into the orbit, compressing and displacing the right lateral rectus muscle and optic nerve. The lesion was expansile, with thinning of the right temporal bone. MRI findings showed the lesion to be hypointense relative to the cerebral gray matter on T1-weighted images, hyperintense on T2-weighted and T2-weighted fluid-attenuated inversion recovery images (**Figures 2B, C**), and isointense on diffusion sequences (**Figure 2D**). Post-contrast T1-weighted MRI demonstrated a vividly enhanced mass with dural involvement (**Figure 2E**).

The patient underwent surgery for both pathological diagnosis and complete tumor removal. A right frontotemporal craniotomy with two burr holes was performed. The tumor was dissected from the dura mater and the infiltrated dura mater showing enhancement on MRI was removed. During the intraoperative evaluation of the intraorbital tumor, it was found that the mass had invaded the temporalis muscle. Intraoperatively, gross resection of the tumor was performed, including partial removal of the superior and lateral orbital walls. Macroscopically, near-total resection was achieved and the adherent parts to the intraorbital tissues were cauterized with electrocoagulation. Cranioplasty was performed using eight titanium screws and four titanium interlink plates. A Simpson grade 2 resection was estimated. The post-operative enhanced MRI scans revealed patchy enhancement within the region that was previously occupied by the lesion, implying the existence of residual disease in the involved dura mater (**Figure 2F**). The postoperative course was favorable, with significant improvement in right-eye vision observed. A 1-year follow-up MRI scan showed no evidence of tumor recurrence.

Histopathological analysis revealed a tumor composed of epithelioid cells and long spindle-shaped cells with some arranged in a swirling pattern. Immunohistochemistry showed positivity for EMA (**Figure 2G**).

The pathology confirmed the diagnosis of a primary intraosseous meningioma of the meningothelial type, classified as WHO Grade I.

## 4 Discussion and conclusion

PEMs account for fewer than 2% of all meningiomas (1). Among these, PIMs, which originate within the bone, are estimated to constitute approximately two-thirds of PEMs (2). According to the 2007 WHO classification, meningiomas are categorized into three grades: benign meningioma (Grade I), atypical meningioma (Grade II), and anaplastic meningioma (Grade III). The treatment strategies and prognoses for these grades differ significantly (3). Meningothelial meningioma is not only the most prevalent pathological variant of PIM but also the most common type among low-risk WHO grade I meningiomas. In contrast, chordoid meningioma, a rare WHO grade II variant, is characterized by its potential for recurrence and clinical aggressiveness. It occurs more frequently in younger individuals and female patients. While intracranial meningiomas are generally slow-growing and benign, prior studies have shown that intracranial meningiomas are associated with a higher malignant profile compared to intradural meningiomas (11% vs. 2%) (5–7). Omofoye et al. (8) also reported no cases of recurrence in patients with WHO grade I PIMs, while WHO grade II cases exhibited a recurrence rate of 33.3%, which was confirmed to be statistically significant. These findings underscore the importance of accurately identifying the pathological grade of PIM preoperatively. Proper grading has critical implications for determining the extent of surgical resection and assessing the patient’s postoperative prognosis.

The etiology of PIMs remains unclear, with several hypotheses proposed. One theory suggests that PIMs originate from multipotent mesenchymal cell precursors (9). Another posits that blood vessels and nerves penetrating the skull may transport arachnoid cap cells to various sites, where they subsequently proliferate (10). Additionally, calvarial meningiomas have been speculated to result from meningothelial cells becoming misplaced and trapped along post-traumatic fracture lines (11). It has also been suggested that PIMs may develop from ectopic meningocytes or arachnoid cap cells embedded in the cranial sutures during the molding of the head at birth (12).

The position and size of the tumor in patients with PIMs significantly determine the clinical presentation. The most common symptom, reported in approximately 68.6% of cases, is a slow-growing painless mass. Other presentations include headache (16.7% of cases) and, more rarely, eye protrusion, blindness, aphasia, hemiparesis, and vertigo. These clinical features show no significant difference compared to those observed in patients with WHO grade I and II meningiomas (8). WHO grade I meningiomas are typically slow-growing and long-standing, causing neurologically relevant clinical symptoms only when the tumor grows large enough to compress the surrounding normal brain tissue. This may explain why the lesion in case 2 was larger than the case 1 lesion in this article.

Accordingly, PIMs can be categorized into three categories based on bone modification: hyperostotic, osteolytic, and mixed. Approximately 59% of cases exhibit hyperostosis, 32% show osteolysis, and mixed features are observed in approximately 6% of cases (13). In this case report, the CT bone window in case 1 revealed a lesion involving parts of the temporal and sphenoid bones, characterized by reduced density, localized cortical bone defects, and partial thickening with increased density of the lateral orbital wall, indicative of mixed features. In contrast, the CT bone window in case 2 demonstrated confined expansive changes in the sphenoid bone, with partial thickening and increased density of the bone cortex, while maintaining continuity, consistent with hyperostotic features.

Studies suggest that benign PIMs typically exhibit expansive growth, whereas malignant PIMs are more likely to present with osteolytic skull lesions and soft-tissue masses (4). Additionally, bone proliferation in benign cases is usually subtle on enhanced imaging, whereas malignant cases often show simultaneous enhancement of bone destruction and surrounding soft-tissue masses. These observations align with the findings in the two cases presented in this case report (14). Therefore, in patients with suspected PIM, significant osteolytic bone destruction observed on CT bone windows, coupled with enhancement of both the involved bone and surrounding soft tissues after gadolinium administration, should raise the possibility of a WHO grade II or higher lesion.

Studies have observed significant differences in MRI features between WHO grade I and II meningiomas, including lobular signs, cystic changes, signal homogeneity, peritumoral edema, tumor-cerebral interface, and homogeneous enhancement. However, some research has indicated that the presence or absence of peritumoral edema and the degree of edema correlated poorly with the site and the size of the tumor, the histological type, and differential diagnosis between malignant and benign (15). Further investigations are needed to clarify these relationships.

Another study suggested that the imaging features of PIMs are correlated with their pathology type. For instance, meningothelial meningiomas, composed of tightly arranged meningeal-like epithelial cells with minimal mesenchyme, no granulomas, and fewer cystic changes, resemble normal brain tissue. On MRI, these tumors typically display isointense signals on T1WI and T2WI, homogeneous enhancement with moderate intensity, and moderate to severe peritumoral edema, which occurs in approximately 66.7% of cases. The imaging features in case 2 in this case report align with these characteristics.

In one study, there were 25 cases of chordoid meningiomas, most of which had regular morphology on MRI, with equal or slightly low signal on T1, equal or slightly high signal on T2, varying degrees of peritumoral edema, and the characteristic “dural tail sign.” These features align with typical meningioma presentations. Case 1 in this case report generally matches these descriptions. More specifically, the lesion appeared to be divided by the dura into two distinct parts with differing morphology and signals. The anterior part was more irregular, with blurred borders, iso-hyper signal intensity on T2WI, and non-enhanced portions around the lesion on contrast-enhanced T1WI, whereas the posterior portion was more irregular, with blurred borders, iso-hyperintense signals on T2WI, and non-enhanced areas on contrast-enhanced T1WI. In contrast, the posterior portion was well-defined, with clear borders, homogeneous hypersignals on T2WI, and homogeneous enhancement on T1WI, along with the “dural tail sign.”

This discrepancy in morphology and signal within the two portions of the lesion may be linked to its pathological type, as the typical meningioma area appeared mixed with a chordoma-like area, consistent with previous speculations. When a lesion suspected of being PIM demonstrates such morphological and signal discrepancies between separated portions, a chordoid type should be considered. Given the diverse intracranial manifestations, the radiographic diagnosis of PIM can be challenging. Several intracranial lesions may need to be considered in the differential diagnosis:

1. Cranial metastases: Typically associated with a known primary tumor, cranial metastases often present as osteolytic lesions invading the brain parenchyma and are surrounded by significant peritumoral edema.
2. Eosinophilic granuloma: Most commonly seen in young individuals, these lesions are usually non-expansive and osteolytic, often invading both the inner and outer plates, particularly the outer plate. They are relatively soft in texture and exhibit less enhancement compared to PIMs.
3. Solitary fibrous tumor: Often displaying a “dural tail sign” on enhancement, solitary fibrous tumors can be mistaken for PIMs. However, they typically exhibit osteolytic destruction. Originating from the meningeal mesenchyme, their MRI signal resembles muscle and shows marked enhancement.

In case 1, which involves a chordoid meningioma (WHO grade II) with high aggressiveness, the differential diagnosis becomes even more critical. Beyond the intracranial lesions mentioned above, it is essential to distinguish it from chordoma-like tumors, such as:

1. Chordoma: Most commonly located in the sella region, clivus, or sacrococcygeal vertebrae, chordomas are prone to bone destruction and often form bone or intraosseous masses.
2. Myxoid chondrasarcoma: Characterized by patchy high-density calcification and ossified shadows on CT, this tumor typically shows homogeneous high signals with sharp borders on T2WI. Bleeding and necrosis are common features.

Accurate differentiation between these entities is crucial for effective diagnosis and treatment planning.

In summary, PIM should be highly suspected when a lesion exhibits both inward and outward growth centered on the skull, accompanied by a “dural tail sign” after gadolinium administration, and when its CT and MRI features resemble those of typical meningiomas. Moreover, if osteolytic calvarial lesions are associated with a soft-tissue component and the affected skull demonstrates enhancement in parallel with the surrounding soft tissue, or if MRI reveals deep lobing, heterogeneous enhancement, and an irregular tumor-brain interface, a classification of WHO grade II or higher should be considered. Additionally, if a lesion contains two regions with distinct morphology and signals, the possibility of a chordoid-type meningioma should be explored.

## 5 Conclusion

CT and MRI are valuable tools for evaluating the nature of PIMs in patients with varying pathological grades.

## Data Availability

The original contributions presented in the study are included in the article/supplementary material. Further inquiries can be directed to the corresponding author.
